# Properties of Biochar Derived from Tea Waste as an Alternative Fuel and Its Effect on Phytotoxicity of Seed Germination for Soil Applications

**DOI:** 10.3390/ma15248709

**Published:** 2022-12-07

**Authors:** Barbora Tunklová, Lukáš Jeníček, Jan Malaťák, Michal Neškudla, Jan Velebil, František Hnilička

**Affiliations:** 1 Faculty of Agrobiology, Food and Natural Resources, Czech University of Life Sciences Prague, Kamýcká 129, 165 00 Prague, Czech Republic; 2 Faculty of Engineering, Czech University of Life Sciences Prague, Kamýcká 129, 165 00 Prague, Czech Republic

**Keywords:** biomass, biofuel, biochar, calorific value, tea waste, phytotoxicity

## Abstract

Tea waste as a potential biofuel and bio fertilizer was analyzed. Samples were collected from various tea species and torrefied to five different temperatures. All samples were analyzed for their proximal composition and calorific value. From the results, stoichiometric properties were calculated. A phytotoxicity test was performed, and the germination index was measured. Tea waste torrefied at 350 °C may be suitable biofuel reaching the calorific value of 25–27 MJ kg^−1^, but with quite a high share of ash, up to 10%, which makes its use technically challenging and may lead to operating issues in a combustion chamber. The same biochar may be a suitable fertilizer for increasing the germination index, therefore, applicable to the soil. The non-torrefied sample and the sample treated at 250 °C are not suitable as fertilizers for being toxic. The total phenolic content in waste black tea was reduced from 41.26 to 0.21 mg g^−1^, depending on the torrefaction temperature. The total flavonoid content was also reduced from 60.49 to 0.5 mg g^−1^. The total antioxidant activity in the non-torrefied sample was 144 mg g^−1^, and after torrefaction at 550 °C, it was 0.82 mg g^−1^. The results showed that black tea waste residues have the potential for further use, for example, in agriculture as a soil amendment or as a potential biofuel.

## 1. Introduction

The limited reserves and aim to lower the European dependency on fossil fuels, the desire to protect nature by decreasing greenhouse gas emissions, and waste production elimination by introducing the circular economy approach are few of the many motivating factors for looking for a new natural resource, especially in the area of biomass waste. In the circular economy concept, waste should re-enter the production flow as recyclable or compostable material or as a fuel [1]. When used as a fuel, dry methods are mostly used, such as direct combustion [2,3], gasification [4], and pyrolysis [5], where low-temperature pyrolysis is an important treatment for low-energy waste [6,7,8].

One of the materials that are globally consumed and leaves a large share of reusable waste is tea. Tea is a dried leaf-infused beverage that is derived from the leaves of *Camellia sinensis* L. It is the world’s second most popular non-alcoholic beverage, with global production of almost 6 million tons in 2017 [9]. The worldwide consumption of tea creates a similar problem to coffee. Over 90% of the tea material is left as waste after its consumption [10]. Tea leaf brewing waste is a lignocellulosic biomass waste that is produced during the tea brewing process. Tea brewing waste can be produced through domestic or industrial processes. According to Taşar, based on statistical reports, the reserves of tea brewing waste are high and should be evaluated [11].

Tea waste is already being researched in many areas of reutilization. A large share of research deals with potential medical applications for tea waste since tea has shown a variety of health benefits, such as the alleviation of metabolic syndrome, anti-tumor effects, and enhanced immunity [12]. Tea leaves are composed of cellulose, hemicellulose, lignin, polyphenols, and proteins, whereas the rich polyphenol content of tea is responsible for various health benefits such as antioxidative, anti-inflammatory, anticarcinogenic, anti-tumor, cardio-protective and several other biological activities [13]. Following Chaudhuri, black tea extract showed significant inhibition against glucose oxidase-mediated inflammation, both in the exudative and proliferative forms as well in the chronic phase of inflammation [14].

Tea is also used as a water pollution absorbent. Antibiotic contamination and the spread of antimicrobial-resistant bacteria are global environmental issues that can be removed using carbon-based materials, including activated carbon, biochar, etc. The usage of sustainable biochar for the sorption of water contaminants brings economic and environmental benefits [15,16,17,18]. From the perspective of tea waste usage as a fuel resource, biogas and solid fuel production options are being widely discussed. Aksay [19], Manyuchi [20], Ayas [21], Ozarslan [22], and Çaǧlar [23] found the positive impact of tea waste usage as a natural additive when producing biogas.

Tea waste was also evaluated by Mizuno [24] as a material suitable for pellet production. In his results, the final pellet diameter did not affect the material structure composed of cellulose, hemicellulose, and lignin. Pua measured the calorific value and conducted the proximal analysis of a pellet made from tea waste that was produced during tea collection in Malaysia. A pellet made from 100% tea waste exhibited a calorific value of 17.393 MJ·kg^−1^, a durability index of 99.93%, and a pellet moisture content of 9.581%. The results showed that tea waste has great potential to be a resource for fuel pellet production [25]. Intagun [26] measured the applicability of tea waste used as an additive to other materials for pellet production, for example, sugarcane bagasse. The results showed that the addition of tea waste positively increased the bulk density and calorific value of sugarcane bagasse. Tea waste proved to be a suitable natural additive [26]. However, a high level of ash and deposit-forming elements were detected in fuel pellets produced when using agricultural residues [27,28].

Tea waste used as biofuel enhancement when torrefied is still an area to discover, with a lot of prospects. Only a few similar research inquiries have been carried out so far. Cai investigated the thermal behavior of refused tea leaves and waste tea with TGA under oxy-fuel and an air atmosphere [29], and Liu showed that the hydrochar pellets had a higher fixed carbon content, elevated heating values, and enhanced mass densities in comparison to raw biomass pellets using hydrothermal carbonization (HTC) combined with pellet production. One of the applications of HTC is the use of tea-activated carbon as an adsorbent [30]. Furthermore, HTC can be used to treat kitchen waste to create a source of raw materials for energy [31].

The tea waste emission factor is also discussed in many articles. Most of the researchers cited similar results indicating that the major emission of tea factor is the final preparation of a hot beverage when boiling the water. The results suggest that the total impact of tea is equal to 12.45 kg CO_2_ eq. kg^−1^ of dry tea for large-scale and 12.08 kg CO_2_ eq. kg^−1^ for small-scale production. The main contributor is tea consumption which is responsible for 85% of the impact because of the electricity used to boil water [32,33]. Based on Cichorowski, cradle-to-gate of 1 kg of Darjeeling tea is between 7.1 and 25.3 kg CO_2,_ depending on the cultivation method, energy sources used, or mode of transportation. The cradle-to-grave for one litter organic Darjeeling tea is about 0.15 kg CO_2_. The largest share, 51%, makes up the use phase, which is clearly dominated by the boiling of water [34]. As per Liang, the total emissions are distributed to energy use in tea processing (41%), fertilizer production (31.6%), and soil emissions (26.7%), leading to an average of 10.8 kg CO_2_ kg^−1^ of processed tea [35].

The application of biochar can improve soil properties and store huge amounts of carbon previously captured from the atmosphere by photosynthesis [36]. Many studies have found that biochar helps the soil to better retain air, water, and nutrients dissolved in it. Biochar is a porous, slowly decomposing material. It also binds minerals, making it easier for soil micro-organisms to access these substances [37]. Biochar can be used directly as a soil additive, or it can be added to compost to improve the composting process and the quality of the final product. If it is added directly to the soil, it is better to saturate the biochar with fertilizer at first. The nutrient saturation prevents the temporary extraction of nutrients from the soil into the biochar, and the nutrients are slowly released from the biochar, so there is no risk of a single overdose [38,39].

The torrefaction of tea waste is less cited in the literature, and only a few articles investigate the use of tea waste as a fuel or soil amendment. The goal of this paper is to evaluate the applicability of tea waste of different grinding fraction sizes and its biochar as an alternative fuel or as a soil amendment.

The hypothesis for this paper is that tea waste can be used as an alternative to fossil fuels and as an alternative to soil amendments. The second hypothesis is that the torrefaction of the material increases the energy yield and enhances the material properties.

## 2. Materials and Methods

Tea waste, which is the leftover from tea beverage preparation (TEA), was chosen as a material for this research. Specifically, was used black tea (*Camellia sinensis* L. Kuntze.) that was collected by one household over 4 months and dried naturally in the sun, reaching final moisture of 7.06%. For tea preparation, usually, the leaves and leaf buds were used.

Tea waste was sorted by particle size into three groups: 0–3 mm, 3–6 mm, and 6–9 mm (Table 1) and was then torrefied in a Thermogravimetric analyzer LECO TGA 701 at a rate of 10 °C. min^−1^ and maintained there for another 60 min at the listed level of temperature (Table 2).

### 2.1. Proximate and Ultimate Analysis

To understand the material components and properties, a proximate and ultimate analysis was performed for each sample. Elemental composition, moisture, and ash content were measured in the first stage of the analysis. The calorific value was measured in the second stage.

The moisture and ash content were measured by Thermogravimetric analyzer LECO TGA 701 (LECO, Saint Joseph, MI, USA). To define the moisture, 1 g of each sample was dried at 105 °C until constant weight. To measure the ash share, 1 g of each sample was heated with increased oxygen concentrations up to 550 °C until constant weight.

Elements composition was analyzed with the Elemental analyzer LECO CHN628 + S using the LECO instrumental biomass combustion method. Each sample of 0.1 g was burned in oxygen at a temperature of 950 °C to determine the C, H, and N values. Oxygen was calculated up to 100% of the dry sample. The elemental analyzer was regularly calibrated with ethylenediaminetetraacetic acid, rice, and rye flour.

To measure the calorific value, 1 g of each sample was placed in a stainless-steel cup in a secured bomb where the pressure was increased up to 3 MPa at a reference temperature of 28 °C. The bomb was then placed inside the isoperbolic calorimeter LECO AC600 where the material was ignited by a cotton thread, and the whole combustion process was controlled. The device was calibrated with benzoic acid.

### 2.2. Stoichiometric Analysis

To better control the combustion operations in power plants and compare theoretical and actual combustion results, stoichiometric calculations must be performed.

Stoichiometric calculations also help in comparing the results among other materials [40]. Following the Technical standards for biomass combustion, the oxygen reference amount was set to 10% [41].

These calculations determine:

Net calorific value based on material moisture, where the calorific value was calculated based on the results of a proximate and ultimate analysis for each sample. The calorific value is calculated as the gross calorific value *Q_s_* (kJ kg^−1^) and the net calorific value *Q_i_* (kJ kg^−1^) [41]. In this article, calorific value figures are used.

The theoretical amount of oxygen needed for complete combustion *O_2,min_* (m^3^ kg^−1^) was based on the equation:

(1) $$O_{2,min}=V_{m}\left(O_{2}\right)\cdot \left(\frac{C}{M\left(C\right)}+\frac{H}{M\left(2\cdot H_{2}\right)}+\frac{S}{M\left(S\right)}-\frac{O}{M\left(O_{2}\right)}\right)$$

where *C*, *H*, *S*, and *O* are contents of carbon, hydrogen, sulfur, and oxygen in the sample (% wt.), respectively; *V_m_(O_2_)* = 22.39 m^3^ kmol^−1^ is the molar volume of oxygen gas at normal conditions; and *M(X)* (kg kmol^−1^) are molar masses of hypothetical species *X* that combine with *O*_2_.

The theoretical amount of dry combustion air *L_min_* (m^3^ kg^−1^) that was determined from the equation:

(2) $$L_{min}=O_{2,min}\cdot \frac{100}{C_{atm}\left(O_{2}\right)}$$

where *C_atm_*(*O*_2_) = 23.20% wt. is a mass concentration of oxygen in the air.

The theoretical amount of dry flue gas *v_fg,min_* (kg kg^−1^) was calculated using the equation:

(3) $$v_{fg,min}=\frac{V_{m}\left(CO_{2}\right)}{M\left(C\right)}\cdot C+\frac{V_{m}\left(SO_{2}\right)}{M\left(S\right)}\cdot S+\frac{V_{m}\left(N_{2}\right)}{M\left(N_{2}\right)}\cdot N+\frac{C_{atm}\left(N_{2}\right)}{100}\cdot L_{min}$$

where *V_m_(X)* (kg kmol^−1^) was the molar mass of flue gas components, and *C_atm_(N*_2_*)* = 75.474% wt. is the concentration of *N*_2_ in air.

The theoretical amount of emission concentrations of *CO*_2*,max*_ (kg kg^−1^) was based on the equation:

(4) $$CO_{2,max}=\frac{M\left(C\right)\cdot C}{V_{m}\left(CO_{2}\right)\cdot v_{fg,min}}$$

Volumetric amounts of combustion products:

(5) $$v\left(CO_{2}\right)=\frac{V_{m}\left(CO_{2}\right)}{M\left(C\right)}\cdot C+\frac{C_{atm}\left(CO_{2}\right)}{100}\cdot L$$

(6) $$v\left(SO_{2}\right)=\frac{V_{m}\left(SO_{2}\right)}{\left(M\right)\left(S\right)}\;$$

(7) $$v_{N_{2}}=\frac{V_{m}\left(N_{2}\right)}{M\left(N_{2}\right)}\cdot N+O\frac{C_{atm}\left(N_{2}\right)}{C_{atm\;\;}{\left(O_{2}\right)}_{2,min}}\;$$

The calorific value *Q_i_* of original water content *W* is recalculated to calorific value *Q_in_* at target the water content of *W_t_* using the formula:

(8) $$Q_{in}=\frac{100-W_{t}}{100-W}\cdot \left(Q_{i}+24.42\cdot W\right)-24.42\cdot W_{t}$$

where *W_t_* (% wt.) is the water content in the target sample, *W* is the water content in the original sample (% wt.), and *Q_i_* is the calorific value of the original water content.

### 2.3. Phytotoxicity Test

The phytotoxicity test of *Lepidium sativum* L. was performed by the department of Botany and Plant Physiology, Czech University of Life Sciences Prague. An aqueous extract was prepared from the torrefied material according to the method previously described by [42]. The toxicity of biochar aqueous extracts was evaluated on seeds of garden cress (*Lepidium sativum* L.). Thirty seeds were placed in a Petri dish lined with one sheet of filter paper previously moistened with test solution (5 mL). Distilled water was used as the control. Five Petri dishes were prepared for each testing variant. The Petri dishes were germinated in an incubator for 48 h in the dark at 25 °C.

The germination index, which is an indicator of biochar toxicity, was calculated using:

(9) $$GI=\frac{k_{v}\cdot l_{v}}{k_{k}\cdot l_{k}}\cdot 100\;\;\;\;\;\;\;\left(\%\right)$$

*k_v_*—germination of the sample;

*k_k_*—germination of the control variant;

*l_v_*—average root length of the sample (mm);

*l_k_*—average root length of the control (mm).

Root lengths were also measured for each sample. A germination index of at least 50% is required for use in the soil [43]. A seed was considered physiologically germinated if the radicle was longer than 2 mm.

### 2.4. Preparation of the Extract

For the determination of total phenolics, flavonoids, and total antioxidant activity, methanol extracts were first prepared. The methanol extract of black tea (*Camelia sinensis* L.) was prepared according to the Czech Pharmacopoeia [44]. The 0.2 g sample was weighed and poured in 5 mL of 70% methanol. Subsequently, the extract was shaken continuously and heated to 70 °C for 25 min. Then, the samples were centrifuged (2350× *g*) for 10 min, and the supernatant was stored in a 10 mL volumetric flask. This extraction procedure was repeated once more. Subsequently, the extracts were mixed and filled up to a final volume of 10 ml. Each variant of methanol extract was repeated 3 times at 10 ml. Each variant of methanol extract was implemented in three repetitions.

#### 2.4.1. Determination of the Total Phenolic Content

Total phenolic content (TPC) was determined according to the methodology described by Singleton and Rossi [45] with slight modifications using methanol extract and Folin-Ciocalteu reagent (Sigma-Aldrich, Saint Louis, MO, USA). Absorbance samples were measured on a UV-Vis spectrophotometer (Evolution 210, Thermo Fisher Scientific, Waltham, MA, USA) at a wavelength of 760 nm. The content was calculated as gallic acid equivalents (GAE mg g^−1^ DW).

#### 2.4.2. Determination of the Total Flavonoid Content

Total flavonoid content (TFC) was determined by the colorimetric method with aluminum chloride (Lachner, Neratovice, Czech Republic) according to the methodology previously described by Chia-Chi et al. [46] with slight modifications, using methanolic extracts. Absorbance samples were measured at a wavelength of 415 nm. The total flavonoid content was calculated from a calibration curve, and the result was expressed as quercetin equivalents (QE mg g^−1^ DW).

#### 2.4.3. Determination of the Total Antioxidant Activity

The total antioxidant activity (TAA) was determined using the phosphomolybdenum method, according to Subhasree [47], with slight modifications. The sample tubes were incubated in a water bath at 95 °C for 90 min, then cooled to room temperature. The absorbance of the solution was measured at 695 nm using a spectrophotometer against a blank. TAA is expressed as ascorbic acid equivalents (AAE mg g^−1^ DW). All samples for each variant were repeated three times independently.

### 2.5. Statistical Analysis

A statistical evaluation of the experiment was made using the analysis of variance (ANOVA) with the Tukey test. Statistical analyses were performed using STATISTICA 12.0 CZ software (Statsoft, Tulsa, OK, USA).

## 3. Results and Discussion

### 3.1. Proximate and Ultimate Analysis

There is a big impact of torrefaction on the elemental analysis of the analyzed sample of tea waste. The moisture level of the material decreased from 7.06% wt. for TEA0 down to 0.76% wt. for TEA250 and slowly increased for higher torrefaction temperatures up to 6.29% wt. for TEA550. Materials torrefied to a higher temperature seem to be better moisture absorbents.

With the temperature increase, the hydrogen and oxygen share declined. The hydrogen share decreased from 5.20% wt. for TEA0 to 2.04% wt. for TEA550, and the oxygen share decreased from 34.76% wt. for TEA0 to 3.91% wt. for TEA550.

Elements that increased their concentration with a temperature rise were mainly carbon and nitrogen. Carbon, the main source of combustion heat of the material, increased its concentration from 47.01% wt. for TEA0 to 73.68% wt. for TEA550. The nitrogen share increased from 1.79% wt. for TEA0 to the highest level for TEA300 with a 2.56% wt. share and back to 2.21% wt. for TEA550.

As per Sermyagina, in his results, green tea contains 53.14% wt. of carbon (52.93% wt. for black tea) for TEA0 equivalent. Sermyagina measured the hydrogen share at green tea at 6.17% wt. (6.21% wt. black tea) for the TEA0 equivalent, again slightly more than in our research. On the other hand, Sermyagina measured a lower share of oxygen in the material, green tea 31.56% wt. (32.61% wt. black tea) in comparison to our 34.76% wt. for TEA0 [48].

The undesired part of the fuel, ash, increased its concentration with the rise in temperature, from 4.11% wt. for TEA0 to 11.76% wt. for TEA550. In comparison to other materials, ash share is quite large for tea waste [40,49]. A detailed elemental analysis can be seen in Table 3.

**Figure 1:**
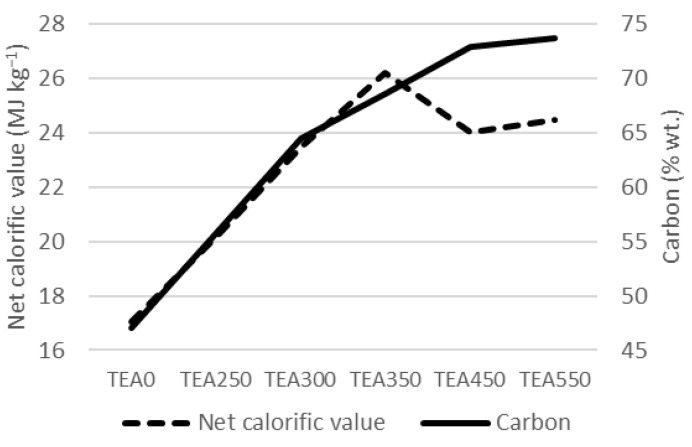
Carbon and net calorific value mutual influence.

From Figure 1, we can clearly see the mutual influence of carbon share and the calorific value of the material. Up to TEA350, a higher share of carbon leads to a higher calorific value of the material. Above the temperature of 350 °C, the material starts to degrade, and the calorific value decreases, even though the carbon share keeps increasing.

In the analysis by Sermyagina, the calorific value is equal to 20.39 MJ kg^−1^ for green tea and 20.26 MJ kg^−1^ for black tea. A bit higher value follows the higher content of carbon [48].

**Figure 2:**
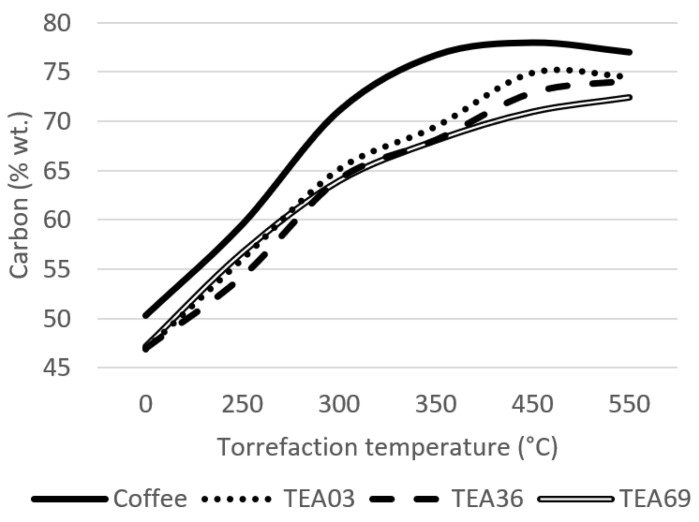
Carbon share of the material.

**Figure 3:**
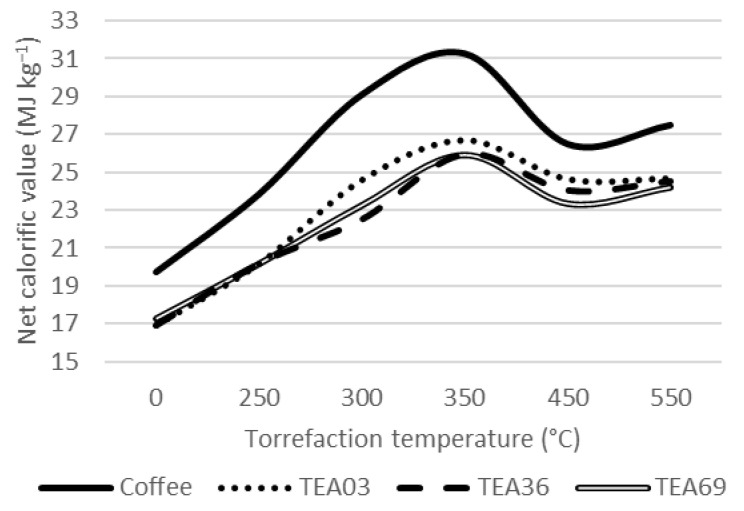
Net calorific value of the material.

**Figure 4:**
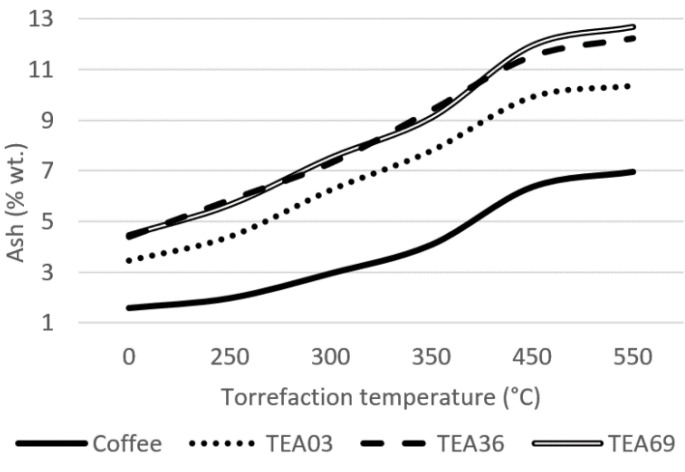
Ash share of the material.

A more detailed analysis was performed for samples divided by particle size, TEA03, TEA36, and TEA69. The results were compared with the spent coffee ground (SCG) to show the difference among similar materials considering the final utilization and preparing of the beverage. Results are shown in Figure 2, Figure 3 and Figure 4.

With the torrefaction temperature increase, the carbon share for the spent coffee ground is slightly higher than for tea samples, which are maintained on very similar levels (Figure 2). TEA03 sample showed the highest carbon share from tea samples at the temperature of 450 °C. The reason for this difference between spent coffee grounds and tea is that spent coffee grounds can manage higher temperatures before the material starts degrading, above 450 °C, due to lower concentration of ash negatively affecting the net calorific value [42].

All tea samples also maintain a very similar level when analyzing the calorific value of the material. As having a slightly higher carbon share, TEA03 showed a slightly higher calorific value at the temperature of 450 °C. The spent coffee ground, in comparison, showed a higher calorific value to tea samples, especially for temperatures of 300 °C and 350 °C. As mentioned before, the reason for this difference is that the material degradation starts at a higher temperature for SCG than for tea samples leading to high concentration of ash for TEA samples (Figure 3).

The ash level is high for tea samples when compared to other materials, e.g., spent coffee grounds. As the ash is the undesired part of the material, its high concentrations are considered negative. The highest share of ash was analyzed for TEA69 at the temperature of 550 °C with 12.69% wt. spent coffee ground contained 6.95% wt. of ash at the same level of torrefaction. TEA03 contained the lowest share of ash of tea samples for all temperatures; considering the highest share of carbon and highest calorific value, TEA03 torrefied at 350 °C is the most suitable adjustment of tea waste for usage as a fuel from a proximate perspective. The reason for higher ash concentrations of tea waste is due to being part of the herbaceous biomass family.

### 3.2. Stoichiometric Analysis

To better understand the fuel combustion behavior under different conditions, various stoichiometric analyses were performed.

**Figure 5:**
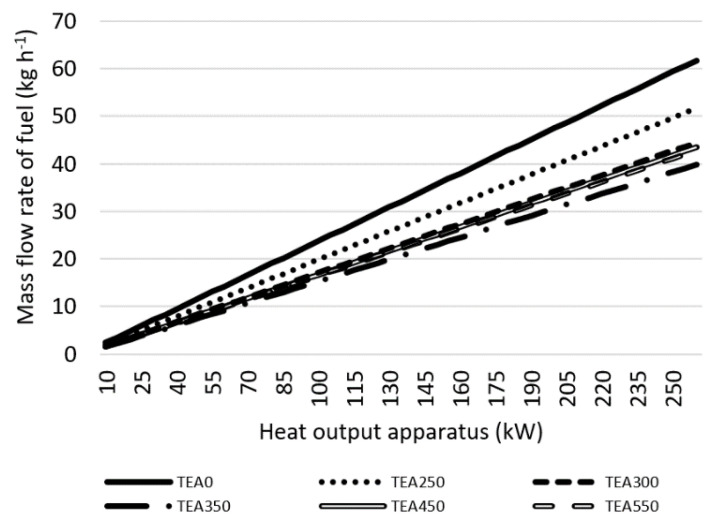
Mass flow rate of fuel fed into combustion chambre to cover the desired heat output of tea waste.

To increase the heat output of the combustion chamber, more fuel needs to be led inside. Figure 5 shows the mass flow needed for a certain heat output. The amount of fuel needed varies per torrefaction level of material. To cover the need for 260 kW of a combustion chamber, 39.81 kg h^−1^ of TEA350 fuel needs to be filled in every hour, but if the material is not torrefied, 61.72 kg h^−1^ of TEA0 would need to be filled in to cover the same heat output.

**Figure 6:**
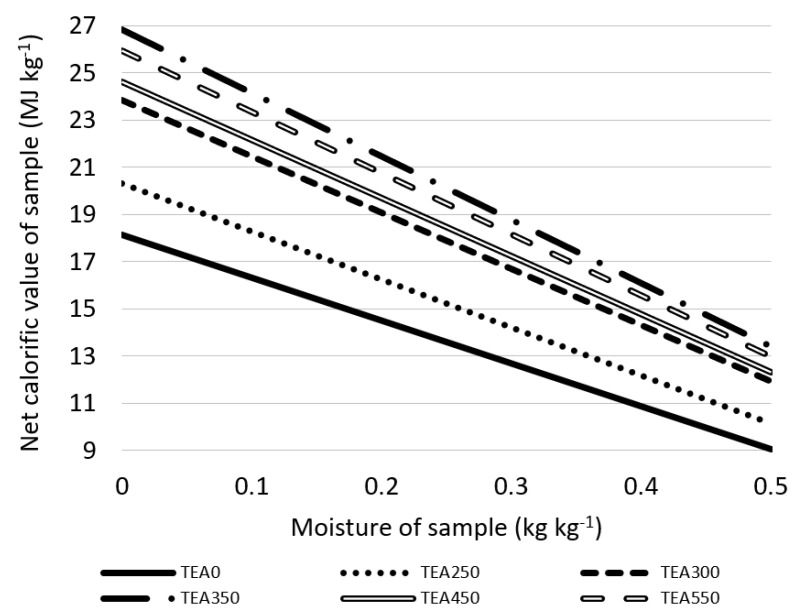
Influence of moisture on calorific value of tea waste.

Material moisture is another factor of fuel efficiency; higher moisture signifies a lower calorific value of material and, therefore, a larger amount to be used to cover the same heat output. The influence between moisture and calorific value is shown in Figure 6. TEA0, material with the lowest calorific value from the analyzed samples, contains 18.13 MJ kg^−1^ when there is no moisture but only 9.06 MJ kg^−1^ if moisture is at 50% wt. TEA350, material with the highest calorific value, contains 26.82 MJ kg^−1^ when there is no moisture and only 13.41 MJ kg^−1^ if moisture is at 50% wt.

**Figure 7:**
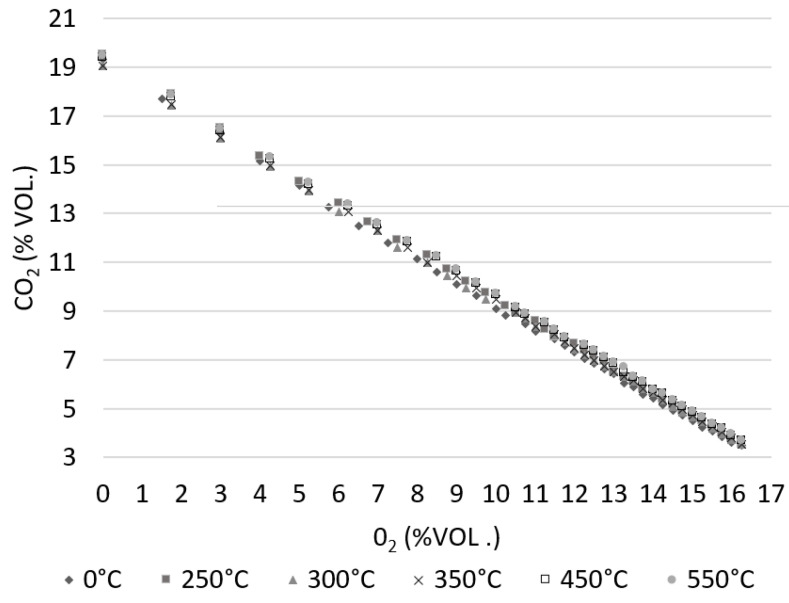
Mutual influence of oxide and carbon dioxide.

The dependence between O_2_ and CO_2_ for all analyzed samples is shown in Figure 7. The results are very similar to all analyzed samples. Higher CO_2_ concentrations are visible for TEA250, which signifies a higher combustion efficiency of the combustion plant and is therefore desired.

The dependence between O_2_ and CO_2_ is:

(10) y=−0.901x+17.618

**Figure 8:**
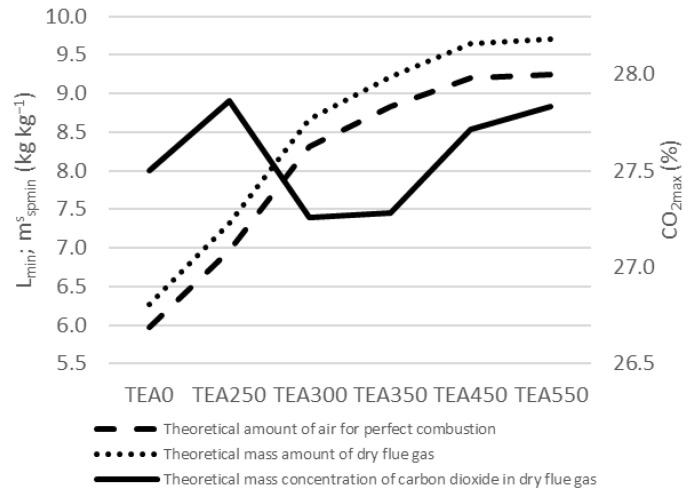
Mass combustion of tea waste.

The theoretical combustion air amounts for perfect combustion are shown in Figure 8. The theoretical amount of air for perfect combustion *L_min_* (kg kg^−1^) increased together with the torrefaction degree up to TEA350. There is a need for 5.97 kg of air for 1 kg of TEA0 to combust or 9.25 kg of air for 1 kg of TEA550. The theoretical mass amount of dry flue gas $[m_{spmin}^{s}\left(kg\;kg^{-1}\right]$ has the same progress with the highest amount for TEA550.

**Figure 9:**
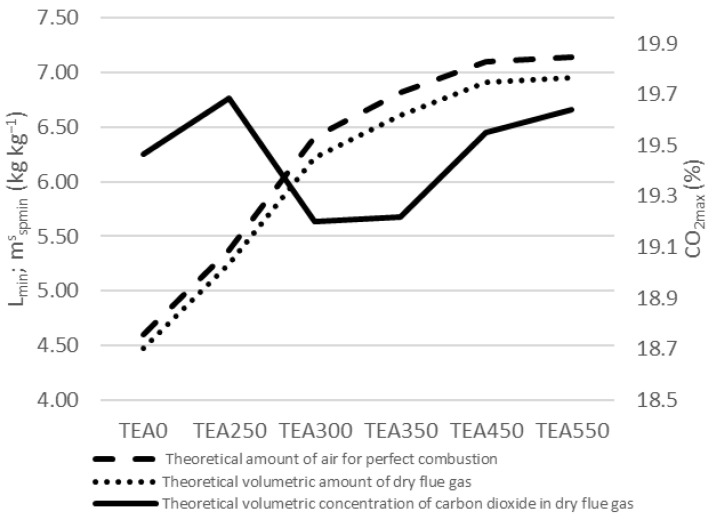
Volumetric combustion of tea waste.

The share of CO_2_ mass concentration in dry flue gas is at the highest level for TEA250 and then decreases for TEA300 and TEA350. CO_2_ mass concentration in dry flue gas is again at a higher level for TEA450 and TEA550. The share varies among 27.2% and 27.9% of dry flue gas: [$CO_{2max}\;\left(\%\;wt.\right)]$. Very similar numbers are also in Figure 9, showing the theoretical volumetric combustion.

The final calculations of the stoichiometric combustion processes in the diffusion combustion fields have a direct dependence on the elemental composition of the treated tea residue samples. The results of the stoichiometric analysis indicate the behavior during real combustion where increased emission concentrations can be avoided as determined in CO and NOx emission measurements for wood fuels [50] and herbaceous biomass [51], but also in the combustion of food industry wastes where significant reductions in emission concentrations were observed [52].

### 3.3. Phytotoxicity Test

**Figure 10:**
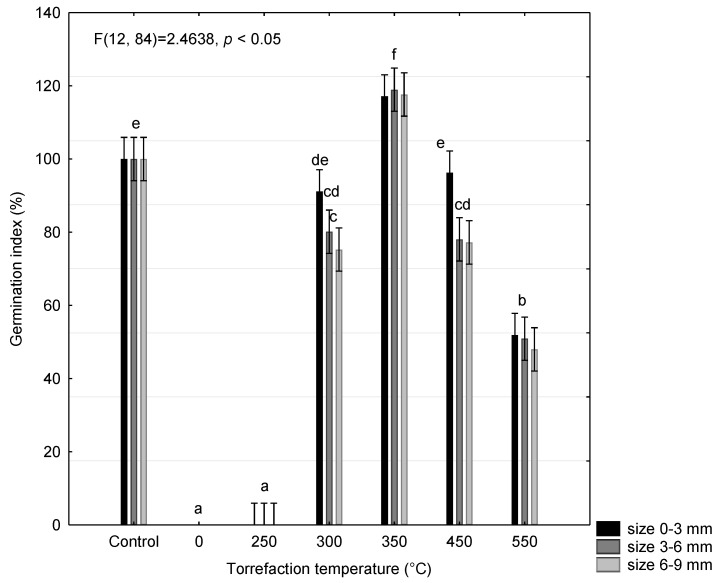
Phytotoxicity affects different grinding fraction sizes of tea aqueous extracts on the germination of *Lepidium sativum* L. seeds after 48 h. Data are expressed as means of five independent bioassays (five replicates for each concentration (aqueous extracts) per bioassay) ± SE. The different letters (a–e) indicate significant differences between treatment effects when compared to the control (ANOVA, Tukey’s test, *p* < 0.05).

As for spent coffee grounds [42], tea waste torrefaction is also applicable as a possible soil amendment. Figure 10 compares the germination index of the tea control sample with analyzed tea samples after torrefaction. In the non-torrefied sample, the index of germination was 0%. The sample torrefied at 250 °C also showed phytotoxic effects. Tea in the form of crude plant waste is characterized by a strong phytotoxic effect, associated, among other things, with its content of flavonoids, catechins, polyphenols, tannins, and other secondary metabolisms [53,54]. According to Rezaeinodehi [55], organic compounds present in spend tea waste may inhibit plant germination or growth.

The phytotoxicity decreased with an increasing torrefaction temperature. The sample at 350 °C ranged from 117 to 119% of the germination index, depending on the size of the grinding fraction. The germination of biochar from tea was also studied by Borgohain [56], who found very similar results. A positive effect of torrefaction on germination was also found by Remay [57]. For samples at 450 °C and 550 °C, as temperature increased, the germination index values decreased.

Grinding coarseness (0–9 mm) does not have a significant effect on the seed germination index, as seen in the graph. From the results, it can be concluded that tea waste can be used as a soil additive, especially the TEA350 torrefaction. Moreover, it is an effective way to recycle bio-waste in a useful way.

Detailed total phenolics (TPC), flavonoids (TFC), and total antioxidant activity (TAA) can be seen in Table 4. It is clear that the values of total phenolic compounds ranged from 41.26 mg g^−1^ to 0.21 mg g^−1^. The highest flavonoids were found for non-torrefied materials, 60.49 mg g^−1^. The total antioxidant activity of black tea waste ranged from 144 mg g^−1^ to 0.82 mg g^−1^, depending on the torrefaction temperature, with the highest value measured at non-torrefied materials.

The results show that increasing temperature pyrolysis decreases the content of all secondary metabolites, as are phenols, flavonoids, antioxidants, and many other organic compounds, and these substances can negatively affect seed germination, as the previous graph shows. In general, the obtained results revealed that the waste from the processed black tea contained appreciable amounts of phenolic and flavonoids compound, as well as high antioxidant activity. Results were in agreement with the statements that were reported by Kopjar, Abdeltaif, and Rahman [58,59,60]. However, it has been found that black tea contains fewer phenolics and flavonoids than green tea [61].

## 4. Conclusions

From the proximal and stoichiometric analysis, tea waste can be considered a suitable fuel additive if torrefied to the level of 350 °C. At this temperature, the calorific value of the material reaches 25–27 MJ kg^−1^, keeping the ash content below 10%. A high ash share is a big disadvantage of tea waste biofuel at all levels of torrefaction compared to other materials. The process of how to remove the excess ash would need to be investigated if burned in big amounts. When comparing tea waste per particle size, the sample with 0–3 mm in diameter showed the highest calorific value and the lowest share of ash compared to other tea samples. Fossil fuel calorific value is very similar, up to 23–28 MJ kg^−1^.

The results of the phytotoxicity test show that tea samples that have not been torrefied and samples torrefied at 250 °C are toxic and unsuitable for soil application. Heat treatment at higher temperatures decomposes substances that have a negative effect on germination. The sample treated at 350 °C showed the highest germination index for all grinding fraction sizes. This biochar can be applied as a soil amendment. It had the best germination parameters in the phytotoxicity test. Furthermore, based on the results, it was found that the non-torrefied material contained the highest content of total phenolics, flavonoids, and total antioxidant activity. With temperature increase, these secondary metabolites, which have a negative effect on seed germination, were decreased.

The first hypothesis can be considered correct for the option of tea waste used as a fuel but not as a soil amendment due to the high level of toxicity of the material. The second hypothesis is correct for both applications; as a fuel, tea waste needs to be used with caution due to high ash share, and as a soil amendment, tea waste had the best germination parameters when torrefied at 350 °C.

## Figures and Tables

**Table 1:** List of samples by particle size.

Tea Waste Sample	Description
TEA03	Tea particle size 0–3 mm
TEA36	Tea particle size 3–6 mm
TEA69	Tea particle size 6–9 mm

**Table 2:** List of samples by torrefaction temperature.

Tea Waste Sample	Particle Size	Final Temperature
TEA0	Average of all	Non-torrefied
TEA250	Average of all	250 °C
TEA300	Average of all	300 °C
TEA350	Average of all	350 °C
TEA450	Average of all	450 °C
TEA550	Average of all	550 °C

**Table 3:** Proximate and ultimate analysis for tea waste sample. Values are means ± SE (*n*  =  3). Different letters (a–f) indicate significant differences based on Tukey’s test (*p* < 0.05).

Tea Waste Sample	Carbon (% wt.)	Hydrogen (% wt.)	Oxygen (% wt.)	Nitrogen (% wt.)	Sulfur (% wt.)	Ash (% wt.)	Moisture (% wt.)
TEA0	47.01 ± 0.28 ^b^	5.20 ± 0.06 ^a^	34.76 ^f^	1.79 ± 0.1 ^a^	0.08 ± 0.03 ^a^	4.11 ± 0.06 ^a^	7.06 ± 0.07 ^e^
TEA250	55.67 ± 0.32 ^c^	5.36 ± 0.07 ^a^	30.46 ^e^	2.34 ± 0.06 ^a^	0.10 ± 0.25 ^a^	5.31 ± 0.10 ^ab^	0.76 ± 0.23 ^b^
TEA300	64.40 ± 0.38 ^d^	4.83 ± 0.07 ^e^	19.25 ^d^	2.56 ± 0.02 ^a^	0.12 ± 0.23 ^a^	7.02 ± 0.06 ^bc^	1.81 ± 0.36 ^c^
TEA350	68.54 ± 0.38 ^e^	4.14 ± 0.07 ^d^	13.29 ^c^	2.55 ± 0.03 ^a^	0.12 ± 0.23 ^a^	8.77 ± 0.20 ^cd^	2.60 ± 0.42 ^a^
TEA450	72.92 ± 0.44 ^a^	3.01 ± 0.10 ^c^	7.57 ^b^	2.49 ± 0.08 ^a^	0.12 ± 0.28 ^a^	11.14 ± 0.17 ^de^	2.76 ± 0.79 ^a^
TEA550	73.68 ± 0.50 ^a^	2.04 ± 0.11 ^b^	3.91 ^a^	2.21 ± 0.04 ^a^	0.11 ± 0.35 ^a^	11.76 ± 0.12 ^e^	6.29 ± 0.92 ^d^

**Table 4:** Total phenolics (TPC), flavonoids (TFC), and total antioxidant activity (TAA) of the methanol extract from black tea. Values are means ± SE (*n*  =  3). Different letters (a–d) indicate significant differences based on Tukey’s test (*p* < 0.05).

Tea Waste Sample	TPC (mg g^−1^)	TFC (mg g^−1^)	TAA (mg g^−1^)
TEA0	41.26 ± 0.88 ^d^	60.49 ± 2.61 ^d^	144.00 ± 12.88 ^d^
TEA250	11.37 ± 0.16 ^c^	16.50 ± 0.50 ^c^	42.61 ± 0.71 ^c^
TEA300	7.56 ± 0.10 ^b^	10.16 ± 0.21 ^b^	29.79 ± 0.57 ^bc^
TEA350	1.80 ± 0.03 ^a^	3.39 ± 0.04 ^a^	6.96 ± 0.12 ^ab^
TEA450	0.32 ± 0.1 ^a^	0.97 ± 0.02 ^a^	1.66 ± 0.05 ^a^
TEA550	0.21 ± 0.03 ^a^	0.50 ± 0.03 ^a^	0.82 ± 0.03 ^a^

## Data Availability

The data not directly presented in the article will be made available on request.

## References

[1] European Environment Agency Closing the Loop—An EU Action Plan for the Circular Economy COM/2015/0614 Final. https://www.eea.europa.eu/policy-documents/com-2015-0614-final.

[2] Malaták J., Jevic P., Gürdil G.A.K., Selvi K.Ç. (2008). Biomass Heat-Emission Characteristics of Energy Plants. AMA Agric. Mech. Asia Afr. Lat. Am..

[3] Malaták J., Velebil J., Bradna J., Gendek A., Tamelová B. (2020). Evaluation of Co and NoxEmissions in Real-Life Operating Conditions of Herbaceous Biomass Briquettes Combustion. Acta Technol. Agric..

[4] Sutton D., Kelleher B., Ross J.R.H. (2001). Review of Literature on Catalysts for Biomass Gasification. Fuel Process. Technol..

[5] Akhtar A., Krepl V., Ivanova T. (2018). A Combined Overview of Combustion, Pyrolysis, and Gasification of Biomass. Energy Fuels.

[6] Bożym M., Gendek A., Siemiątkowski G., Aniszewska M., Malaťák J. (2021). Assessment of the Composition of Forest Waste in Terms of Its Further Use. Materials.

[7] Tamelová B., Malaťák J., Velebil J. (2018). Energy Valorisation of Citrus Peel Waste by Torrefaction Treatment. Agron. Res..

[8] Tamelová B., Malaťák J., Velebil J., Gendek A., Aniszewska M. (2021). Energy Utilization of Torrefied Residue from Wine Production. Materials.

[9] Vivek V. Global Market Report: Tea | International Institute for Sustainable Development. https://www.iisd.org/publications/report/global-market-report-tea.

[10] Akbayrak S., Özçifçi Z., Tabak A. (2020). Activated Carbon Derived from Tea Waste: A Promising Supporting Material for Metal Nanoparticles Used as Catalysts in Hydrolysis of Ammonia Borane. Biomass Bioenergy.

[11] Taşar Ş. (2022). Thermal Conversion Behavior of Cellulose and Hemicellulose Fractions Isolated from Tea Leaf Brewing Waste: Kinetic and Thermodynamic Evaluation. Biomass Convers. Biorefin..

[12] Sheikhzadeh N., Nofouzi K., Delazar A., Oushani A.K. (2011). Immunomodulatory Effects of Decaffeinated Green Tea (*Camellia Sinensis*) on the Immune System of Rainbow Trout (*Oncorhynchus Mykiss*). Fish Shellfish Immunol..

[13] Debnath B., Haldar D., Purkait M.K. (2021). Potential and Sustainable Utilization of Tea Waste: A Review on Present Status and Future Trends. J. Environ. Chem. Eng..

[14] Nag Chaudhuri A.K., Karmakar S., Roy D., Pal S., Pal M., Sen T. (2005). Anti-Inflammatory Activity of Indian Black Tea (Sikkim Variety). Pharmacol. Res..

[15] Krasucka P., Pan B., Sik Ok Y., Mohan D., Sarkar B., Oleszczuk P. (2021). Engineered Biochar—A Sustainable Solution for the Removal of Antibiotics from Water. Chem. Eng. J..

[16] Ahsan M.A., Katla S.K., Islam M.T., Hernandez-Viezcas J.A., Martinez L.M., Díaz-Moreno C.A., Lopez J., Singamaneni S.R., Banuelos J., Gardea-Torresdey J. (2018). Adsorptive Removal of Methylene Blue, Tetracycline and Cr(VI) from Water Using Sulfonated Tea Waste. Environ. Technol. Innov..

[17] Khalil U., Bilal Shakoor M., Ali S., Rizwan M., Nasser Alyemeni M., Wijaya L. (2020). Adsorption-Reduction Performance of Tea Waste and Rice Husk Biochars for Cr(VI) Elimination from Wastewater. J. Saudi Chem. Soc..

[18] Shakoor M.B., Bibi I., Niazi N.K., Shahid M., Nawaz M.F., Farooqi A., Naidu R., Rahman M.M., Murtaza G., Lüttge A. (2018). The Evaluation of Arsenic Contamination Potential, Speciation and Hydrogeochemical Behaviour in Aquifers of Punjab, Pakistan. Chemosphere.

[19] Aksay M.V., Ozkaymak M., Calhan R. (2018). Co-Digestion of Cattle Manure and Tea Waste for Biogas Production. Int. J. Renew. Energy Res..

[20] Manyuchi M.M., Mbohwa C., Muzenda E. Biogas and Bio Solids Production from Tea Waste through Anaerobic Digestion. Proceedings of the International Conference on Industrial Engineering and Operations Management.

[21] Ayas N., Esen T. (2016). Hydrogen Production from Tea Waste. Int. J. Hydrog. Energy.

[22] Özarslan S., Abut S., Atelge M.R., Kaya M., Unalan S. (2021). Modeling and Simulation of Co-Digestion Performance with Artificial Neural Network for Prediction of Methane Production from Tea Factory Waste with Co-Substrate of Spent Tea Waste. Fuel.

[23] Çaǧlar A., Demirbaş A. (2001). Hydrogen-Rich Gaseous Products from Tea Waste by Pyrolysis. Energy Sources.

[24] Mizuno S., Ida T., Fuchihata M., Namba K. (2016). Effect of Specimen Size on Ultimate Compressive Strength of Bio-Coke Produced from Green Tea Grounds. Mech. Eng. J..

[25] Pua F.L., Subari M.S., Ean L.W., Krishnan S.G. (2020). Characterization of Biomass Fuel Pellets Made from Malaysia Tea Waste and Oil Palm Empty Fruit Bunch. Mater. Today Proc..

[26] Intagun W., Kanoksilapatham W., Maden A., Nobaew B. Effect of Natural Additive on Pellets Physical Properties and Energy Cost. Proceedings of the 2019 IEEE 2nd International Conference on Renewable Energy and Power Engineering, REPE 2019.

[27] Zhang J., Guo Y. (2014). Physical Properties of Solid Fuel Briquettes Made from Caragana Korshinskii Kom. Powder Technol..

[28] Zhang L., Xu C.C., Lei H., Wang H.L., Ning T.T., Hao W., Hu X.D. (2014). Effects of Addition of Various Ingredients during Pelletizing on Physical Characteristics of Green Tea Residue Pellets. Appl. Eng. Agric..

[29] Cai H., Zou H., Liu J., Xie W., Kuo J., Buyukada M., Evrendilek F. (2018). Thermal Degradations and Processes of Waste Tea and Tea Leaves via TG-FTIR: Combustion Performances, Kinetics, Thermodynamics, Products and Optimization. Bioresour. Technol..

[30] Islam M.A., Benhouria A., Asif M., Hameed B.H. (2015). Methylene Blue Adsorption on Factory-Rejected Tea Activated Carbon Prepared by Conjunction of Hydrothermal Carbonization and Sodium Hydroxide Activation Processes. J. Taiwan Inst. Chem. Eng..

[31] Malaťák J., Dlabaja T. (2016). Hydrothermal Carbonization of Kitchen Waste. Res. Agric. Eng..

[32] Azapagic A., Bore J., Cheserek B., Kamunya S., Elbehri A. (2016). The Global Warming Potential of Production and Consumption of Kenyan Tea. J. Clean Prod..

[33] Xu Q., Hu K., Wang X., Wang D., Knudsen M.T. (2019). Carbon Footprint and Primary Energy Demand of Organic Tea in China Using a Life Cycle Assessment Approach. J. Clean Prod..

[34] Cichorowski G., Joa B., Hottenroth H., Schmidt M. (2015). Scenario Analysis of Life Cycle Greenhouse Gas Emissions of Darjeeling Tea. Int. J. Life Cycle Assess..

[35] Liang L., Ridoutt B.G., Wang L., Xie B., Li M., Li Z. (2021). China’s Tea Industry: Net Greenhouse Gas Emissions and Mitigation Potential. Agriculture.

[36] He Y., Yao Y., Ji Y., Deng J., Zhou G., Liu R., Shao J., Zhou L., Li N., Zhou X. (2020). Biochar Amendment Boosts Photosynthesis and Biomass in C3 but Not C4 Plants: A Global Synthesis. GCB Bioenergy.

[37] Kuppusamy S., Thavamani P., Megharaj M., Venkateswarlu K., Naidu R. (2016). Agronomic and Remedial Benefits and Risks of Applying Biochar to Soil: Current Knowledge and Future Research Directions. Environ. Int..

[38] Ding Y., Liu Y., Liu S., Li Z., Tan X., Huang X., Zeng G., Zhou L., Zheng B. (2016). Biochar to Improve Soil Fertility. A Review. Agron. Sustain. Dev..

[39] Dey D., Mavi M.S. (2022). Co-Application of Biochar with Non-Pyrolyzed Organic Material Accelerates Carbon Accrual and Nutrient Availability in Soil. Environ. Technol. Innov..

[40] Malaťák J., Passian L. (2011). Heat-Emission Analysis of Small Combustion Equipments for Biomass. Res. Agric. Eng..

[41] Uhlí a Koks—Stanovení Spalného Tepla.

[42] Jeníček L., Tunklová B., Malat’ák J., Neškudla M., Velebil J. (2022). Use of Spent Coffee Ground as an Alternative Fuel and Possible Soil Amendment. Materials.

[43] Silva M.P., Nieva Lobos M.L., Piloni R.v., Dusso D., González Quijón M.E., Scopel A.L., Moyano E.L. (2020). Pyrolytic Biochars from Sunflower Seed Shells, Peanut Shells and Spirulina Algae: Their Potential as Soil Amendment and Natural Growth Regulators. SN Appl. Sci..

[44] State Institute for Drug Control (2017). Czech Pharmacopoeai.

[45] Singleton V.L., Rossi J.A. (1965). Colorimetry of Total Phenolics with Phosphomolybdic-Phosphotungstic Acid Reagents. Am. J. Enol. Vitic..

[46] Chang C.C., Yang M.H., Wen H.M., Chern J.C. (2020). Estimation of Total Flavonoid Content in Propolis by Two Complementary Colometric Methods. J. Food Drug Anal..

[47] Subhasree B., Baskar R., Laxmi Keerthana R., Lijina Susan R., Rajasekaran P. (2009). Evaluation of Antioxidant Potential in Selected Green Leafy Vegetables. Food Chem..

[48] Sermyagina E., Mendoza Martinez C.L., Nikku M., Vakkilainen E. (2021). Spent Coffee Grounds and Tea Leaf Residues: Characterization, Evaluation of Thermal Reactivity and Recovery of High-Value Compounds. Biomass Bioenergy.

[49] Jenicek L., Neskudla M., Malatak J., Velebil J., Passian L. (2021). Spruce and Barley Elemental and Stochiometric Analysis Affected by the Impact of Pellet Production and Torrefaction. Acta Technol. Agric..

[50] Juszczak M. (2020). Comparison of CO and NOx Concentrations from a 20 KW Boiler for Periodic and Constant Wood Pellet Supply. Environ. Prot. Eng..

[51] Tamelová B., Malaťák J., Velebil J., Gendek A., Aniszewska M. (2022). Impact of Torrefaction on Fuel Properties of Aspiration Cleaning Residues. Materials.

[52] Malaťák J., Velebil J., Malaťáková J., Passian L., Bradna J., Tamelová B., Gendek A., Aniszewska M. (2022). Reducing Emissions from Combustion of Grape Residues in Mixtures with Herbaceous Biomass. Materials.

[53] Chatterjee P., Chandra S., Dey P., Bhattacharya S. (2013). Comparative Study of Allelopathic Effects of Green Tea and Black Tea. Curr. Trends Biotechnol. Pharm..

[54] Bizuayehu D., Atlabachew M., Ali M.T. (2016). Determination of Some Selected Secondary Metabolites and Their Invitro Antioxidant Activity in Commercially Available Ethiopian Tea (*Camellia Sinensis*). Springerplus.

[55] Rezaeinodehi A., Khangholi S., Aminidehaghi M. (2006). Allelopathic Potential of Tea (*Camellia Sinensis* (L.) Kuntze) on Germination and Growth of *Amaranthus Retroflexus* L. and *Setaria Glauca* (L.) P. Beauv. J. Plant Dis. Prot. New Ser..

[56] Borgohain A., Konwar K., Buragohain D., Varghese S., Kumar Dutta A., Paul R.K., Khare P., Karak T. (2020). Temperature Effect on Biochar Produced from Tea (*Camellia Sinensis* L.) Pruning Litters: A Comprehensive Treatise on Physico-Chemical and Statistical Approaches. Bioresour. Technol..

[57] Bourguiba H., Scotti I., Sauvage C., Zhebentyayeva T., Ledbetter C., Krška B., Remay A., D’Onofrio C., Iketani H., Christen D. (2020). Genetic Structure of a Worldwide Germplasm Collection of *Prunus armeniaca* L. Reveals Three Major Diffusion Routes for Varieties Coming from the Species’ Center of Origin. Front. Plant Sci..

[58] Kopjar M., Tadić M., Piližota V. (2015). Phenol Content and Antioxidant Activity of Green, Yellow and Black Tea Leaves. Chem. Biol. Technol. Agric..

[59] Abdeltaif S.A., Sirelkhatim K.A., Hassan A.B. (2018). Estimation of Phenolic and Flavonoid Compounds and Antioxidant Activity of Spent Coffee and Black Tea (Processing) Waste for Potential Recovery and Reuse in Sudan. Recycling.

[60] Rahman M., Jahan I.A., Ahmed S., Ahmed K.S., Roy M., Zzaman W., Ahmad I. (2021). Bioactive Compounds and Antioxidant Activity of Black and Green Tea Available in Bangladesh. Food Res..

[61] Kodama D.H., Gonçalves A.E.d.S.S., Lajolo F.M., Genovese M.I. (2010). Flavonoids, Total Phenolics and Antioxidant Capacity: Comparison between Commercial Green Tea Preparations. Food Sci. Technol..

